# Genetic Structure of the French Red Squirrel Populations: Implication for Conservation

**DOI:** 10.1371/journal.pone.0047607

**Published:** 2012-10-17

**Authors:** Anne Dozières, Jean-Louis Chapuis, Sophie Thibault, Emmanuelle Baudry

**Affiliations:** 1 Muséum National d’Histoire Naturelle, Département Ecologie et Gestion de La Biodiversité, UMR 7204 Conservation des Espèces, Restauration et Suivi des Populations, MNHN-CNRS-P6, Paris, France; 2 Université Paris-Sud, Laboratoire Ecologie, Systématique et Evolution, CNRS-UMR 8079, Orsay, France; Biodiversity Insitute of Ontario - University of Guelph, Canada

## Abstract

The decline of the red squirrel (*Sciurus vulgaris*) in several European countries due to the introduction of the American grey squirrel (*S. carolinensis*) and the predicted arrival of the grey squirrel in France in the near future has lead to the development of a preventative conservation project in this country. In this study, we conducted an extensive survey of mitochondrial DNA variation in French red squirrels using a fragment of the mitochondrial D-loop and we compared the results with previously published data from other European populations. Our main aims were: (1) to determine whether genetically differentiated populations, which could represent prioritized units for conservation purposes, were present in France and (2) to determine whether the French population, which is currently largely undisturbed, could provide information on the postglacial recolonization history of the species. We found that French D-loop haplotypes show almost no tendency to cluster by geographic origin, be it region or country, suggesting that French red squirrels have not been isolated from other populations during an evolutionarily significant period and that they do not constitute an Evolutionary Significant Unit. The French red squirrels showed strong signals of population expansion, the opposite to what is observed in most other European populations, making them of particular interest to study the postglacial expansion history of the species.

## Introduction

The red squirrel *Sciurus vulgaris* is a well-known animal found in many types of coniferous, deciduous and mixed forests of Eurasia [1]. It is a very widespread species, with a range extending from the British Isles in the west throughout the Palaearctic to Hokkaido Island in the east [1], [2]. The red squirrel is currently common throughout much of this range and is listed as “Least Concern” on the IUCN Red List [3]. However, it is of conservation concern in Britain, Ireland and, most recently, Italy, due to a combination of loss or fragmentation of its woodland habitat [4] and interspecific competition with the introduced North American *S. carolinensis* [5]. In these countries, the grey squirrel outcompetes the red squirrel for resources in woodlands, leading to a significant decline in native red squirrel populations, and its complete replacement over large areas in Britain [6], [7], [8], [9].

**Figure 1:**
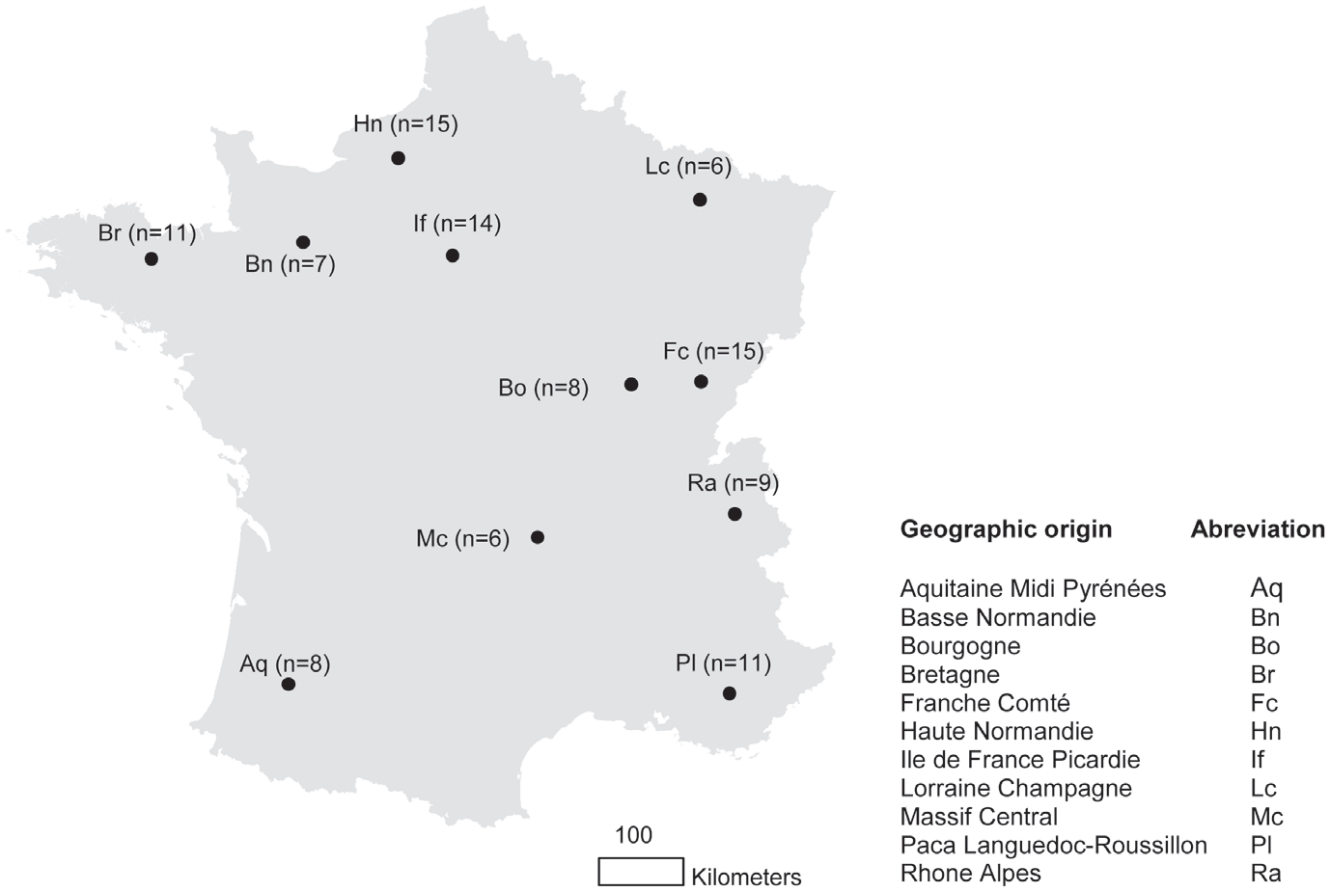
Sampling localities of *Sciurus vulgaris* in France. Locations of the centroide of our sampling sites are indicated in black points, *n* indicates the number of specimens analysed in each locality.

**Table 1:** Summary Statistics of molecular variation and neutrality tests in nine *S. vulgaris* European populations.

Country	Sample size	Haplotype diversity	Nucleotidediversity (%)	Tajima’s D	Fu’s F_S_	Source
France	110	0.976	1.919	−1.614*	−80.41***	This study
Austria	13	0.949	1.974	−0.604	−3.03	Grill *et al.* (2009)
Italy except Calabria	67	0.926	1.849	−0.88	−14.32**	Grill *et al.* (2009)
Great Britain	124	0.764	2.032	0.564	0.18	Hale *et al.* (2004)
Iberia	43	0.764	1.316	0.077	1.99	Grill *et al.* (2009); Hale *et al.* (2004)
Netherland	10	0.733	1.570	1.065	2.29	Hale *et al.* (2004)
Calabria	11	0.182	0.507	−1.896	2.87	Grill *et al.* (2009)
Sweden	13	0.154	0.488	−2.024**	3.01	Hale *et al.* (2004)

* , significant at the 0.05 level;

** , significant at the 0.01 level.

*** , significant at the 0.001 level.

In order to be able to compare values between populations, the 252 pb fragment analyzed by Grill, *et al.* [11] was used for all samples. It corresponds to the positions 1–252 of the fragment analyzed in previous studies [16], [17], [18], [19], [20] and to the positions 111–362 of the fragment analyzed in the present study.

Across its geographical range, *S. vulgaris* has been divided into numerous subspecies primarily based on coat color and morphological variation. The number of these subspecies varies according to the authors: more than 40 have been described by Corbet [10], but the number has been reduced to 17 by Sidorowicz [11]. However, these classifications based on phenotypic variation do not necessarily reflect phylogeography since the observed morphological differences may represent adaptations to local environmental conditions [1]. Recently, a major phylogeographical study by Grill *et al.* [12] has investigated the large scale genetic variation of *S. vulgaris* in its European range by analyzing a sample of 236 individuals from 15 European countries with mitochondrial and microsatellite nuclear markers. While microsatellite genotypes formed three clusters corresponding to three of the subspecies recognized by Sidorowicz [11], mtDNA haplotypes showed an almost complete lack of phylogeographical structure across Europe: only the individuals from the region of Calabria in southern Italy formed a distinct phylogroup, whereas all other European individuals clustered together to form a second unstructured phylogroup. Furthermore, the pattern of mtDNA variability among this second group suggested that a rapid demographic expansion occurred. This genetic structure possibly reflects the evolutionary history of the species during the last glacial maximum. Red squirrels are extremely arboreal and their distribution is closely linked to the distribution of woodland habitat [1]. During Pleistocene glaciations events, deciduous forests were confined into refuges in the Mediterranean peninsula. Forest living species, like red squirrels, have therefore been confined to these regions before recolonizing Europe during the post-glacial reforestation [13]. These forced colonization movements had genetic consequences that affect the present genetic structure of the populations [14].

The red squirrel genetic diversity has also been studied at a finer geographic scale in small isolated populations in Belgium [15], and in Great Britain and north Italy, where *S. vulgaris* populations are threatened by the American grey squirrel [16], [17], [18], [19], [20]. In France, even if predictive models suggest that *S. Carolinensis* will arrive from Italy in the coming decades [21], it has not been observed yet in the country. In this context, the study of French red squirrel is of particular interest. First, a genetic structure study of populations will allow determining whether several Evolutionarily Significant Units (ESUs) are present in France. One of the possible criterions for qualification as an ESU is that a population must show genetic differentiation from other populations at neutral markers, caused by past restriction of gene flow [22]. Identifying such genetically differentiated populations can then be used to define conservation priorities. If the predicted arrival of the grey squirrel results in a decline of French red squirrel populations, prior knowledge of the populations genetic structure will help to set up efficient conservation measures. In their study of European red squirrels, Grill *et al.* [12] detected no genetic differentiation of French individuals but it might well be the consequence of small sample size (n = 6 animals from one locality in France).

**Figure 2:**
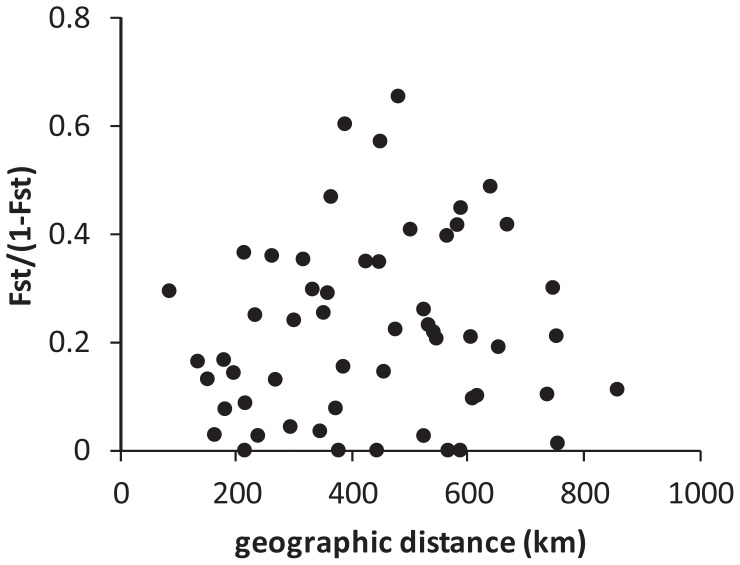
Mantel test of the relationships between genetic divergence and geographic distance among French haplotypes. For 110 French *Sciurus vulgaris* D-loop haplotypes (516 bp fragment) from 11 regions, (F_ST_/(1-F_ST_) is plotted against the distance between the centroides of the sampling sites in kilometers (km).

Second, to our knowledge, there are as yet no fine scale genetic studies on red squirrel genetic diversity in regions where the populations have not been disturbed by interspecific competition, habitat loss or fragmentation. In France, the grey squirrel is currently not present and the total forest area has been increasing since the middle of the 19^th^ century [23], reaching 29% of the territory in 2009 [24], which suggests that habitat loss is not a major threat for the red squirrel in this country. Furthermore, French red squirrel populations have not been modified by translocations of individuals from other geographic areas; existing populations are the result of natural colonization process. In contrast the majority of extant populations of British *S. vulgaris* are of recent continental Europe ancestry, probably the result of translocations of individuals from continental Europe [18]. Phylogeographical characteristics of the French population should therefore reflect ancient geographical and historical events and should be useful in investigating the hypothesis of a postglacial population expansion, that what proposed in previous studies [12], [20].

**Figure 3:**
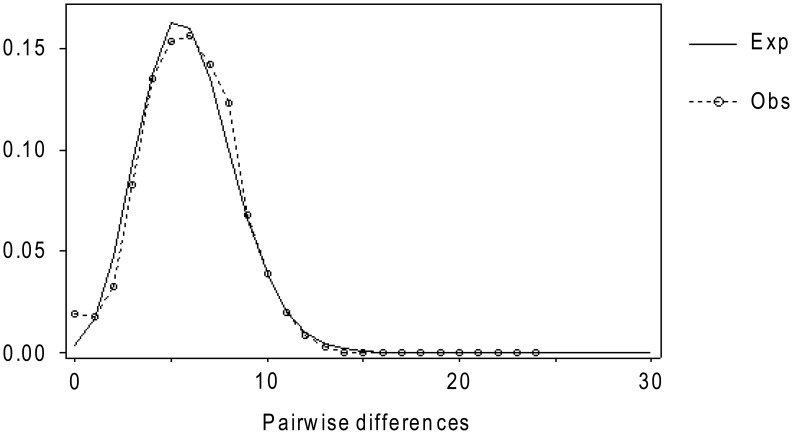
Mismatch distribution for the 110 French *Sciurus vulgaris* D-loop haplotypes (516 bp). The Mismatch distribution is the distribution of the number of pairwise differences among sequences. The expected distribution under a model of population expansion is given as a continuous line, and the observed distribution is given as a dashed line.

In this study, we examined mitochondrial DNA variation among French red squirrels and compared it with those of other European red squirrels with two objectives: to establish whether ESUs are present in France, and to determine whether French red squirrels could be used to improve our understanding of the postglacial expansion history of the species.

## Materials and Methods

### Sample Collection

Tissue samples from dead red squirrels originating from 11 French regions (Figure 1) were collected. Most tissues samples were obtained from animals killed by road traffic, one of the major mortality factors for red squirrels [25]. The specimens were mainly obtained from collaborators of the “Office National des Forêt” between 2009 and 2011. Squirrels were frozen immediately after collection in the field and then an ear biopsy was preserved in 90% ethanol until DNA extraction.

### Ethics Statement

All conducted experiments complied with the current laws of France. In this study we obtained ministerial authorization to transport corpses of this protected species. This derogation was approved by the French “Comité National de Protection de la Nature” (CNPN).

### DNA Extraction, Amplification and Sequencing

DNA was extracted from 50 mg of tissue with a Macherey Nagel Nucleospin tissue kit, following the manufacturer’s protocol. Extracted DNA was resuspended in 100 ml elution buffer. Mitochondrial DNA variation was assayed by the amplification of a 516 bp fragment of the D-loop, matching nucleotide positions 15464–15979 of the published sequence of the complete mtDNA genome of *Sciurus vulgaris* (GenBank Accession no. AJ238588). The fragment was amplified by PCR using the primers Lpro-SQL and SQR-SQR designed by Trizio *et al.* [26]. Each PCR reaction was run in a 20 µl volume containing 1 µl of DNA solution, 400 µM of each dNTP, 1.75 µM of Mg^++^, 1 µM of each primer and 1.25 units of Taq polymerase (Qiagen). Thermocycle conditions were 94°C for 30 s, 48°C for 30 s, and 72°C for 1 min, for a total of 35 cycles. Purified template DNA was sequenced on both strands with the PCR primers, using standard Sanger sequencing techniques. All sequences were proof read and aligned manually, generating a 516 bp alignment for 110 French red squirrels. Sequences were deposited into the GenBank database under accession numbers JX645360 to JX645469.

**Figure 4:**
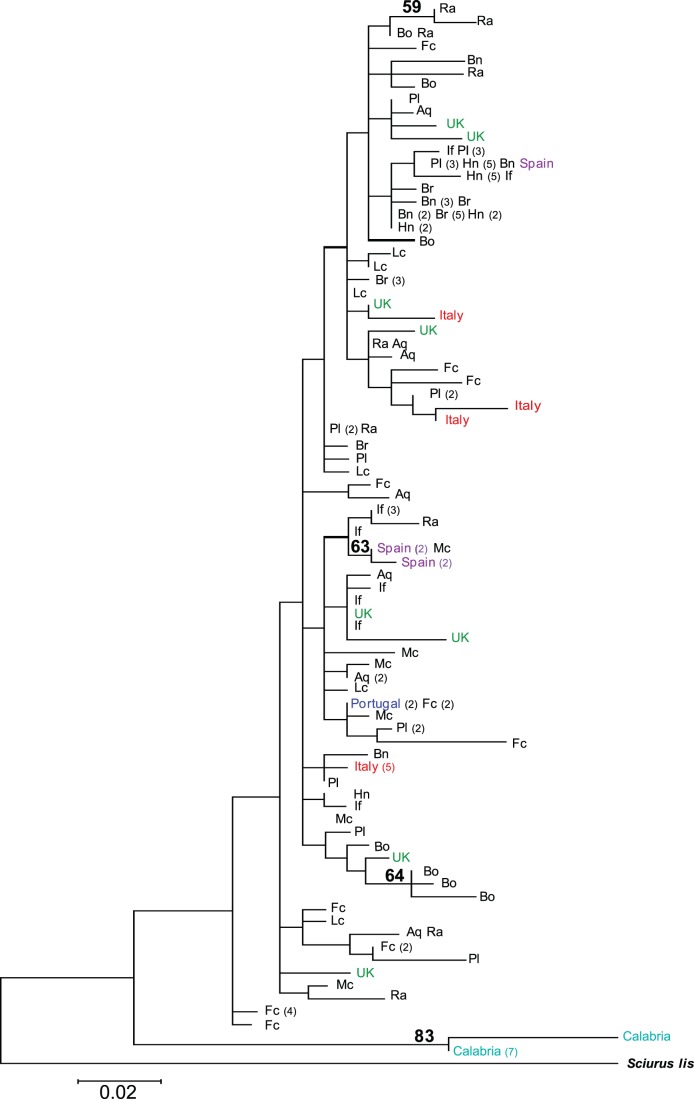
Maximum likelihood tree of French and European *Sciurus vulgaris* D-loop haplotypes (252 bp). Bootstrap values are shown as percentage of 1000 replicates at each node only if they are 50% or greater. The numbers of identical haplotypes per locality are indicated in brackets. Abbreviations indicate the geographical origin of French samples : Aq: Aquitaine Midi Pyrénées; Bn: Basse Normandie; Br: Bourgogne; Fc: Franche Comté; Hn : Haute Normandie; If : Ile-de-France; Lc : Lorraine Champagne; Mc : Massif Central; Pl : Paca Languedoc-Roussillon; Ra : Rhône Alpes.

### Analysis of Sequence Data

We used DNAsp5 to analyze sequence variations (haplotype number, estimation of nucleotide polymorphism), to calculate genetic differentiation using F_ST_, and to perform neutrality tests. To assess past demographic history, we calculated two standard neutrality tests, Tajima’s D [27], which compares two estimators of polymorphism, and Fu’s F_S_ [28], which compares the observed number of haplotypes in a sample to the expected number under neutrality. In the absence of selection, population expansion is indicated by negative values of D and F_S_. The significance of these two statistics tests was estimated by 10 000 coalescent simulations, using DNAsp5. The demographic history of French red squirrels was also inferred by a pairwise mismatch distribution analysis between individuals [29] computed under a population growth-decline model. Multimodal distributions are consistent with demographic stability, while population expansion generates an unimodal pattern [30].

We also used a coalescent approach to validate the patterns of demographic history revealed by the mismatch distributions [31]. We used the program FLUCTUATE from the Lamarc package [32] to test if the data fit a model of exponentially growing or stable populations, estimating the parameter *θ* = *Ne µ* (where *Ne* is the effective population size and *µ* the mutation rate) and *g* (the exponential rate of population growth or decline relative to the neutral mutation rate) [32]. The appropriate transition: transversion ratio (estimated with MEGA5, see below) was used. Ten short MCMC simulations of 200 generations each and two long MCMC simulations of 20,000 generations each were used to explore the solution space. The probability that ‘g’ ≥0, *i.e.* the population has undergone an expansion was determined by using the likelihood surface produced by fluctuation. The analysis was repeated ten times and the mean and standard deviation of *θ* and *g* were calculated from the results of these ten runs.

We tested whether Slatkin’s isolation-by-distance model [33] of increased genetic distance with increased geographic distance between populations is appropriate for our French red squirrels samples. The significance of the Pearson correlation coefficient between genetic differentiation and geographic distance was assessed with a Mantel test using Arlequin v. 3.11 [34].

We used the maximum likelihood method to assess the phylogenetic relationships among *S. vulgaris* haplotypes. Nucleotide sequences of red squirrels from several other European countries were downloaded from GenBank and added to our data set, generating a 252 bp D-loop alignment. To determine which model of nucleotide substitution is the most appropriate for this dataset, we used MEGA version 5 [35] to test hierarchically the effect of unequal base frequencies, different rates between transitions and transversions, different rates between all substitutions, rate variation over nucleotide sites, and presence of invariant sites. The model that best fitted the dataset was then used with MEGA 5 to reconstruct a phylogenetic tree, rooted with a sequence of the Japanese squirrel, *S. lis*, the closest relative of *S. vulgaris* [11], by maximum likelihood analysis with nearest neighbor interchange. The reliability of the tree obtained was examined using 1000 bootstrap replicates.

Finally, in order to compare genetic variability and neutrality test values for our French samples with those obtained for other European samples in previous studies, we calculated these estimators as described above but using only the 252 bp alignment. We did not include populations whose individuals were sampled from a unique location in order to be able to compare values, except for the Calabria population because of its particular interest.

## Results

Sequences of a mtDNA D-loop fragment (516 bp) were successfully determined for 110 French red squirrels. This fragment encompasses the 252 bp and 395 D-loop fragments used in previous studies [12], [18], [20], and largely overlaps the fragment analyzed by Barratt *et al.* [17]. They resulted in 71 different haplotypes. 57 sites were variable, among which 41 were parsimony informative and 16 singletons. Haplotype diversity in the total dataset was high (0.981±0.006 for all individuals). Within population, haplotype diversity ranged from 0.752 in the Hn population to 1 in the Aq, Bo, Mc, Lc and RA population, with a mean value of 0.934. The nucleotide diversity was 0.0117±0.0232 in the total dataset. Within populations, nucleotide diversity ranged from 0.0051 in the Br population to 0.0132 in the FC population, with a mean value of 0.0098.

Substantial genetic subdivision existed among the 11 populations (F_ST_ = 0.167; P<0.001), and between the majority of pairs of populations (not shown). A mantel test performed with the Arlequin software was not significant (*P* = 0.63; Figure 2), indicating that genetic distance does not increase with geographic distance among French red squirrel populations.

Tajima’s *D* and Fu’s *Fs* neutrality tests had significantly negative values (*D* = −1,583; *P*<0.05 and *Fs* = −80.89; *P*<0.0001).The mismatch distribution of the D-loop sequences showed an unimodal distribution (Figure 3) which was almost indistinguishable from the one expected under a population growth-decline model [30].

FLUCTUATE was used to jointly estimate the parameters *θ* and *g*, it found a positive growth rate in the French squirrel population: the most likely estimates for the two parameters were *θ = *0.696*±*0.045 and *g* = 402.1*±*14.8. The probability that the squirrel population has not undergone expansion, *i.e. P*(*g* ≤0 ), was less than 0.01.

The model of nucleotide substitution that best fit the 252 bp alignment of 110 French squirrels and 32 European squirrels sequences was a Hasegawa-Kishino-Yano (HKY85) model [36] with rate heterogeneity among sites (gamma distribution shape parameter of 0.61) and invariant sites (0.58). This model was then used to reconstruct a phylogenetic tree by maximum likelihood analysis. The phylogeny (Figure 4) revealed that haplotypes showed almost no tendency to cluster by geographic origin, be it region or country. The individuals from Calabria in southern Italy seem to be the only lineage that differed significantly from the rest of the sample (bootstrap value of 83). All other haplotypes formed a largely unresolved cluster (almost no bootstrap values above 50, Figure 4).

The comparison of the genetic variability and neutrality test values obtained in different European populations by previous studies (252 bp fragment) showed that samples from Calabria and Sweden had low levels of variation compared to samples from other countries (Table 1). Tajima’s *D* and Fu’s *Fs* neutrality tests are significantly negative for the French population (Table 1), as was observed when analyzing the 516 bp fragment (see above).

## Discussion

We found that French D-loop haplotypes do not show any tendency to cluster by geographic origin, similar to what was described for other countries or in Europe as a whole [12], [17], [18]. This has two possible explanations: (1) the French populations of red squirrel are not genetically differentiated because of high levels of gene flow, or (2) the lack of geographic structure is due to a recent origin from a common refuge, as suggested by Grill *et al.* [12]. Several lines of evidence suggest that the second explanation is very likely the correct one. First, French populations show a high level of genetic differentiation, suggesting that the level of gene flow between population is currently low. This is in agreement with the known relatively low mobility of the species [37], [38]. Second, the mismatch distribution of the D-loop haplotypes is markedly unimodal, which is observed when the genealogy of the sample resembles a star phylogeny, which in turn is observed during demographic expansions [30]. Third, Tajima’s *D* and Fu’s *Fs* neutrality tests have significantly negative values. This indicates that the French D-loop haplotypes show an excess of singletons and an excess of haplotypes, features that are observed following a demographic expansion. Finally, the coalescent simulations performed with FLUCTUATE to estimate the demographic history of the French red squirrel also demonstrated that an expansion took place. Our analysis of French red squirrel therefore strongly supports the hypothesis by Grill *et al.* [12] of a demographic expansion from a glacial refugium.

We observed a high haplotype and nucleotide diversity among French populations, in agreement with the results of previous studies (e.g. [12], [17], [18]. Due to their short generation times and large effective population sizes, rodents usually show a high level of genetic diversity [39]. Moreover, in the case of the red squirrel, the high observed genetic variability is also probably related to the low mobility of the species [37], [38], which leads to population subdivision and can increase global genetic diversity [40]. In order to be able to compare the values obtained for different populations by different studies, summary statistics of genetic variation and neutrality tests for several European populations of red squirrel were calculated using only the 252 pb D-loop fragment analyzed by Grill *et al.* [12] (Table 1). We found that only two populations showed low levels of variation. In Calabria, 11 individuals originating from one area showed only two haplotypes that were very divergent from all other European haplotypes described so far. Grill *et al.* [12] hypothesized that this low variability in haplotypes could be the result of a continuously low population size. In Sweden, a sample of 13 individuals originating from different places distributed over the entire country showed also only two different haplotypes [18]. This low variation can likely be explained by serial founder effects [14] during post-glacial recolonisation of the Scandinavian peninsula [18], as was recently described for Scandinavian brown bears [41]. In contrast, all other populations showed a high level of genetic variability. Surprisingly, the populations of Italy and Iberia, areas that are potential glacial refuges [42], do not present higher genetic variability than the other European populations. This may suggest that the individuals that recolonized Europe after the last glacial maximum may have come from the Balkans or from Asia, though this needs to be confirmed by genetic studies of squirrels from these parts of the distribution. Alternatively, it is possible that Europe was recolonized by squirrels originated from Italy or Iberia and that later bottlenecks reduced the genetic diversity of their populations [12]. Interestingly, the neutrality tests values (Table 1) indicate that the French population of red squirrels is the only one that shows a non ambiguous signal of population expansion, with significantly negative values of both Tajima’s *D* and Fu’s *F_S_* tests, suggesting that it is of particular interest to understand the postglacial expansion history of the species.

Another objective of our study was to establish whether the French red squirrels were genetically distinct from other European populations. As described in previous genetic studies of red squirrels [12], [17], [18], [20], we observed an almost complete absence of geographic partitioning of haplotypes. Haplotypes found in French red squirrels were completely interspersed with those from other countries, and no grouping by region of origin was observed within French haplotypes (Figure 4). This is consistent with traditional morphological classifications under which French red squirrels are classified as *S. v. fuscoater* subspecies, together with the majority of the mainland European populations [11]. This suggests that the French red squirrel population has not been isolated from other populations during an evolutionarily significant period, and that it is not an ESU [22]. However, we also found that almost all the French populations of our sampling were significantly differentiated from each other. It is therefore possible that different French populations have different local adaptations caused by local environmental conditions, which would be of conservation interest. For example, the darker coat coloration frequently observed in mountainous regions could be adaptive [43]. Moreover, while mitochondrial DNA is very useful for detecting ancient events [44], it is not ideal for detecting more recent events. In opposite, microsatellites, with their high mutation rates, are particularly suitable to study recent events [44]. This is probably the reason why divergent results are sometimes reported between mitochondrial and microsatellite markers: for example, the Iberian squirrels were differentiated as a separate group from other European squirrels with microsatellite data but not with mtDNA [12]. It would therefore be very interesting to analyze French squirrels with microsatellites data, in order to detect possible recent isolation processes in France [45].
